# Supplementary material for: Higher-order quantum spin Hall effect in a photonic crystal
Source: Nat Commun. 2020 Jul 28;11:3768. doi: 10.1038/s41467-020-17593-8 (PMC7387344; doi:10.1038/s41467-020-17593-8)
Supplement: Supplementary file 1 — Supplementary Information [file 41467_2020_17593_MOESM1_ESM.pdf]

Supplementary Information for

Higher-order Quantum Spin Hall Effect in a Photonic Crystal

Xie et al.

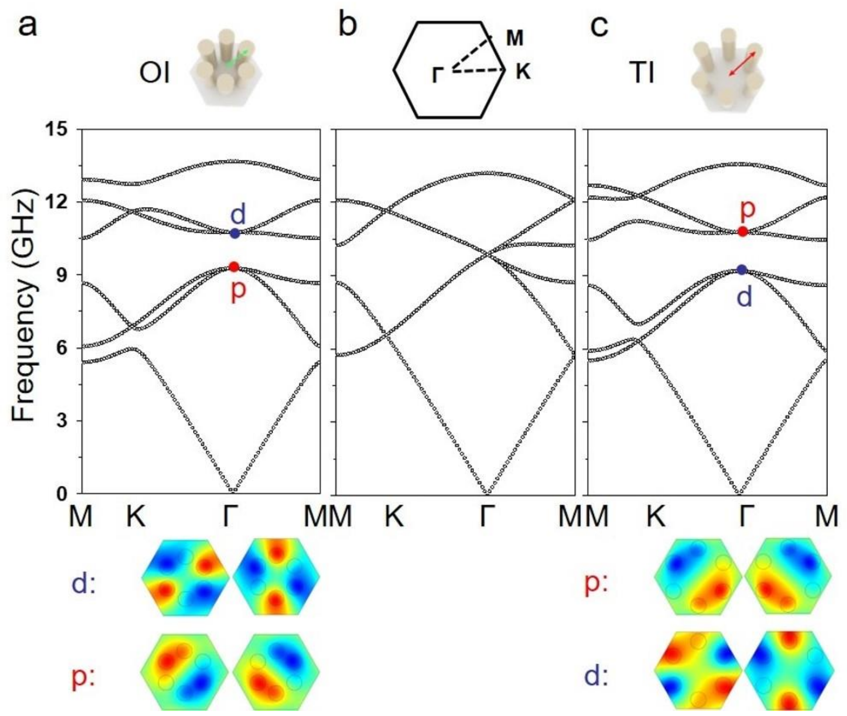

Supplementary Figure 1. | Band inversion and topological phase transition. a The shrunken lattice with a fully gapped band structure. The degenerate point of the second and third (the fourth and fifth) bands are p (d) states. **b** Phase transition point with a four-fold degenerate point. **c** The expanded lattice with an inversed fully gapped band structure. The degenerate point of the second and third (the fourth and fifth) bands are d (p) states.

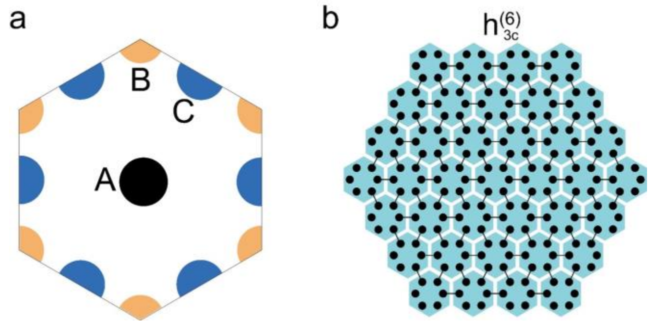

Supplementary Figure 2. | Maximal Wyckoff positions and the primitive generator. a The three mirror symmetry in the unit cell will restrict the Wannier center to be at those high symmetric points (Wyckoff positions) at the unit cell. **b** Primitive generator of our photonic crystals which determine the coupling configuration. The intercell coupling is not depicted here.

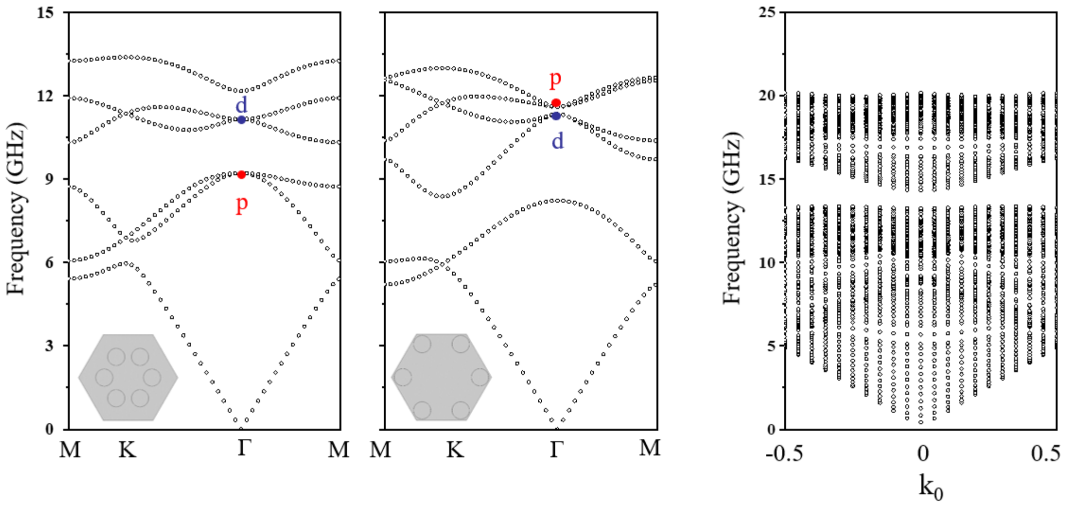

Supplementary Figure 3. | The band structures for $h^{(6)}_{4b}$ case of photonic crystals. The band structures of photonic crystals in $h^{(6)}_{4b}$ configuration with shrunken lattice (left panel), expanded lattice (middle panel), and the projected band structure of two juxtaposed photonic crystals (right panel). The distances between the centers of dielectric rods and the center of the unit cells are 5 mm and 8 mm for the photonic crystals in the left panel and the middle panel respectively with other parameters fixed as those of photonic crystals in Fig 2. For the case with small intra-cell coupling (middle panel), there is no full bandgap in the band structure.

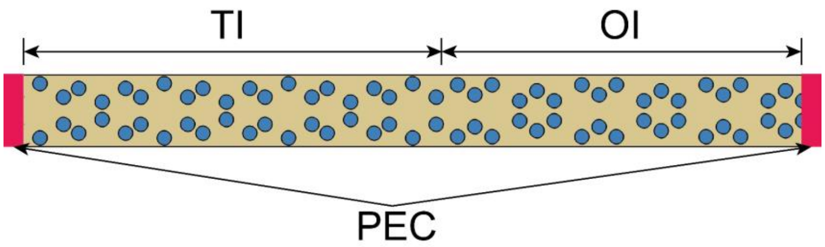

Supplementary Figure 4. | The ribbon structure to obtain the projected band structure. We set the boundary parallel to the interface between the TI and OI as the perfect electric conductor (PEC) boundary (red area) and the boundary perpendicular to the interface between the TI and OI as Floquet periodic boundary (black lines).

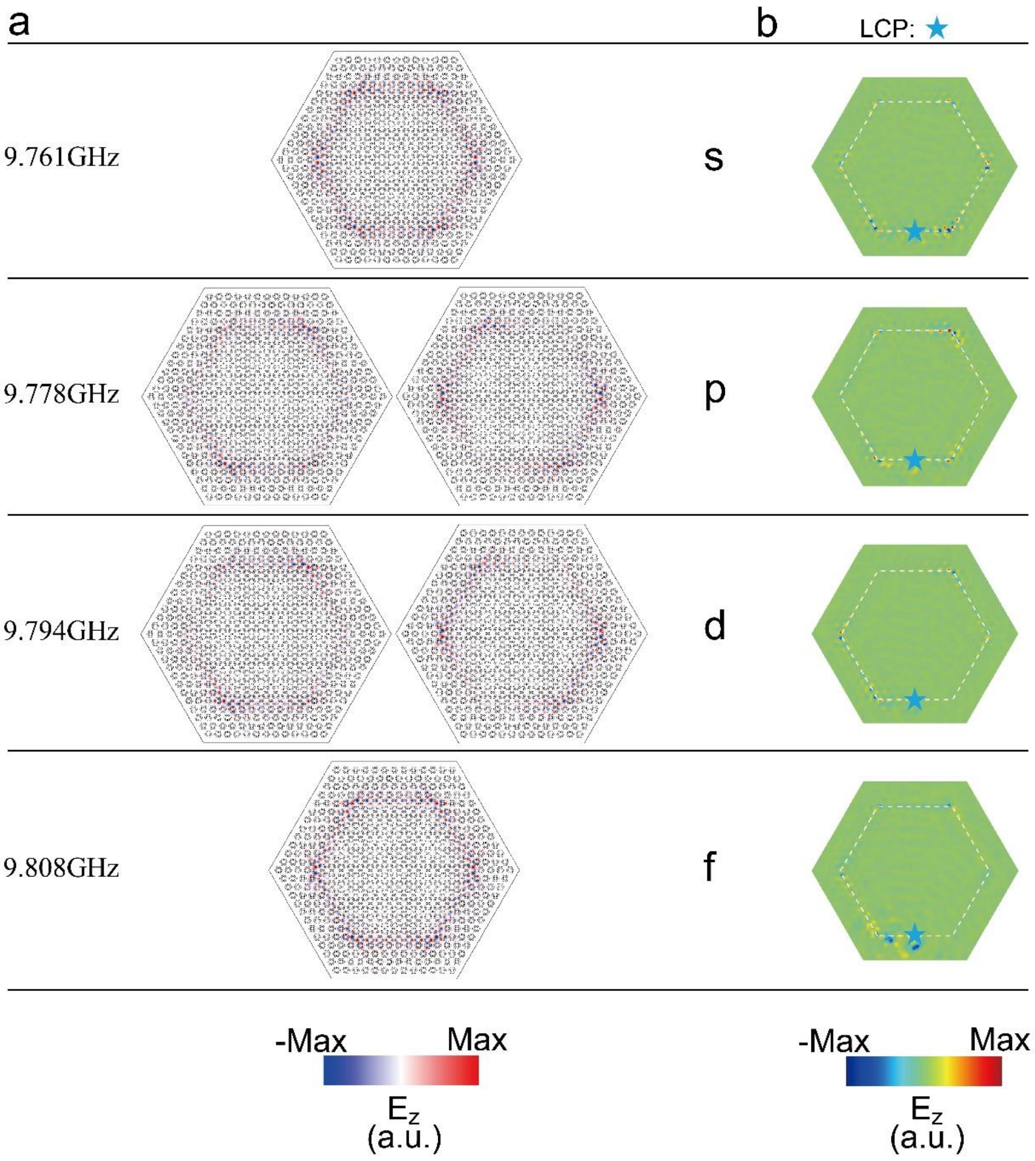

Supplementary Figure 5. | Eigenmodes of the hexagonal metastructure. a The simulated E_z of six eigenmodes of the corner states with a small frequency difference induced by the finite-size coupling. **b** Experimental measured E_z of the corner states with a source located at the interface between SOTI and OI (blue star). We note that for dipole modes and quadrupole modes, the measured field distributions are their linear combinations.

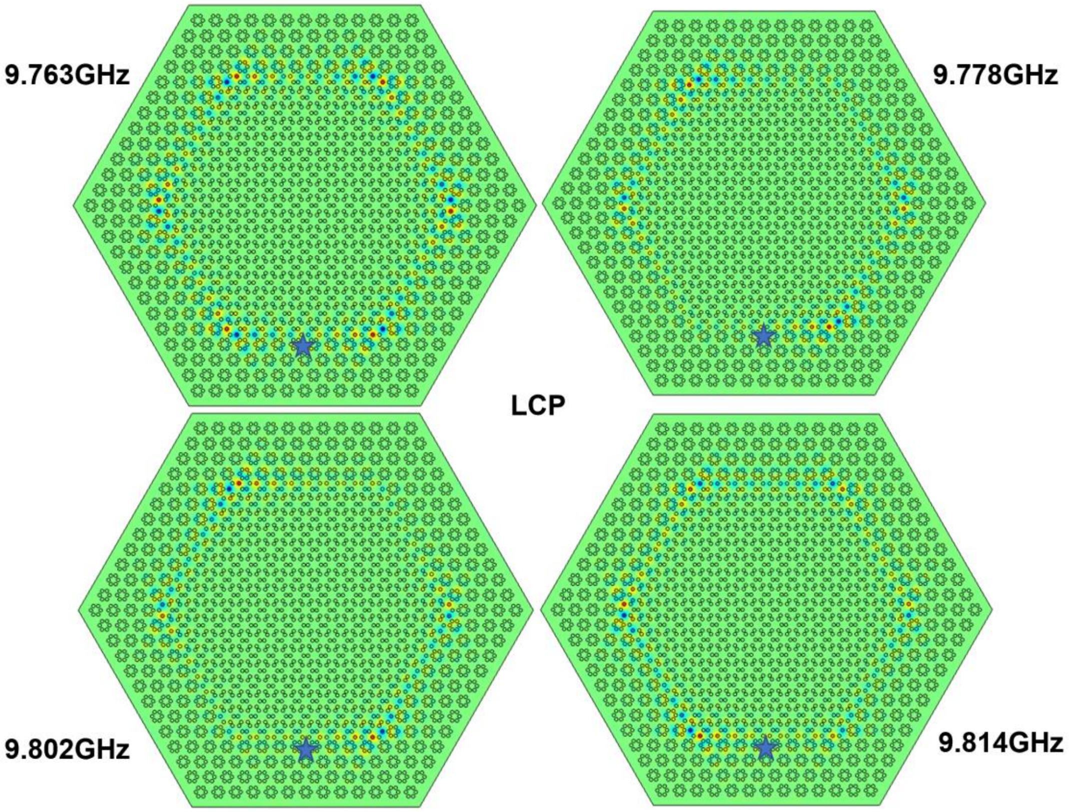

Supplementary Figure 6. | The excited field distribution of all corner states. Due to the closed boundary, the field distribution far away from the source could be much higher than that from adjacent corners to the source.

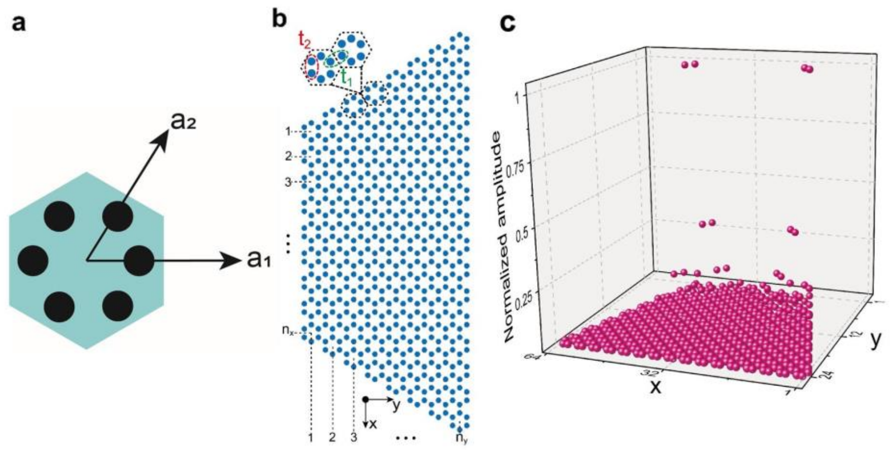

Supplementary Figure 7. | Tight-binding model which mimics our photonic crystals. a, The unit cell of the tight-binding model. The black dots represent the lattice sites. **b,** The finite-size structure of the tight-binding lattice with only nearest-neighbor couplings. Here we choose $n_x = 9$ and $n_y = 8$. **c**, The normalized amplitude of the zero-energy corner states of the tight-binding lattice.

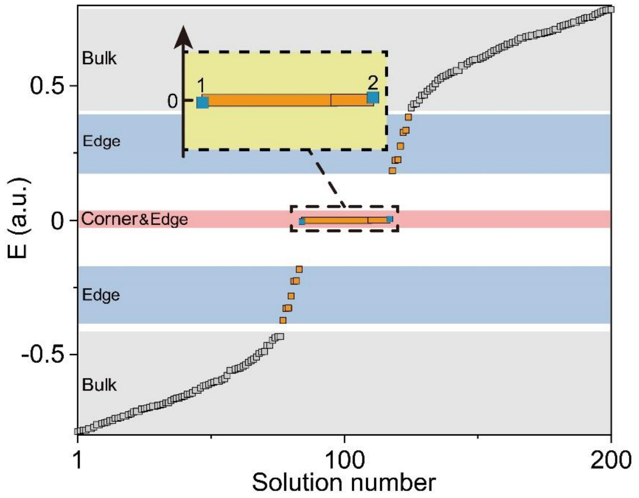

Supplementary Figure 8. | The eigenmode distribution of a finite-size tight-binding model. Bulk states, edge states and corner states are represented by grey, yellow and blue dots respectively. There are two corner states whose energies are slightly split from the zero energy.

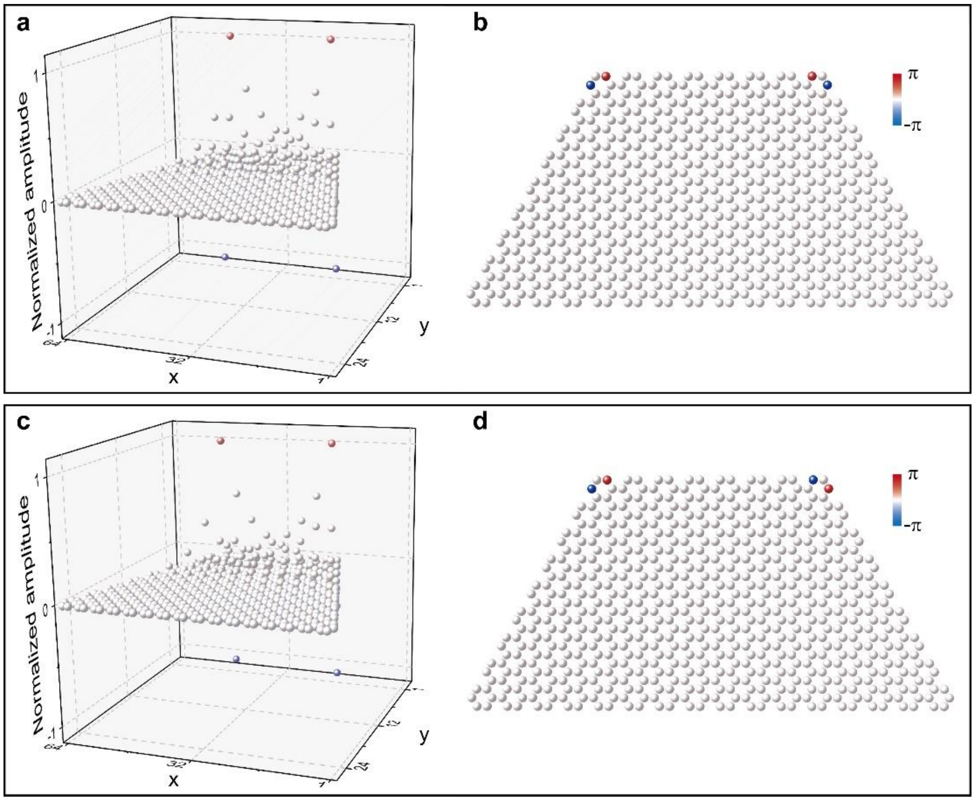

Supplementary Figure 9. | The field distribution with phases of two corner states in the half-hexagonal tight-binding model. The corner state labeled as 1(2) in Supplementary Figure 6 is shown in **a** and **b** (**c** and **d**) respectively. Here we only plot the phases of two corner states.

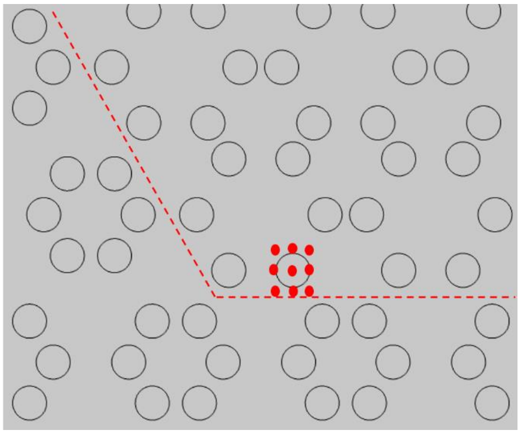

Supplementary Figure 10. | The measured positions of the spectrum for corner states. We take the averaged value of the measured data at nine points around one dielectric rod.

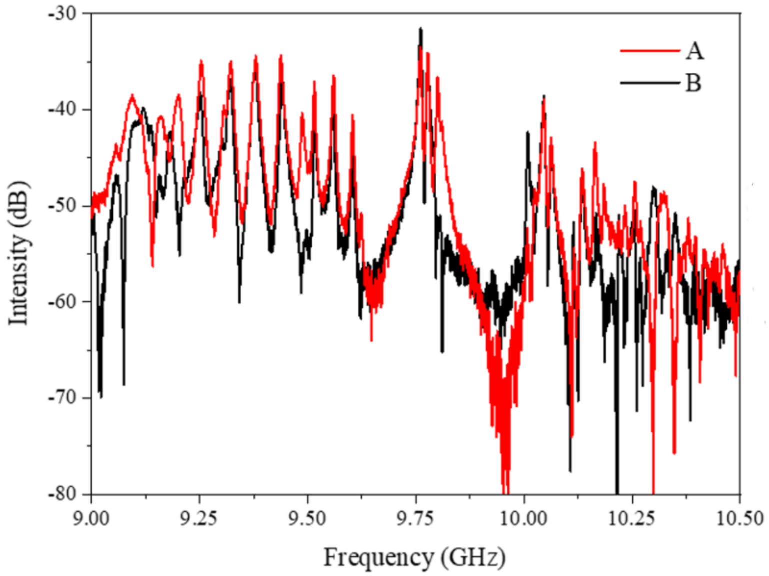

Supplementary Figure 11. | The measured local field intensities at the different corners. We find that the intensity measured at the left corner (represented by the red line) as shown in Fig. 2**e** is almost the same as the one measured at the right corner (represented by the black line).

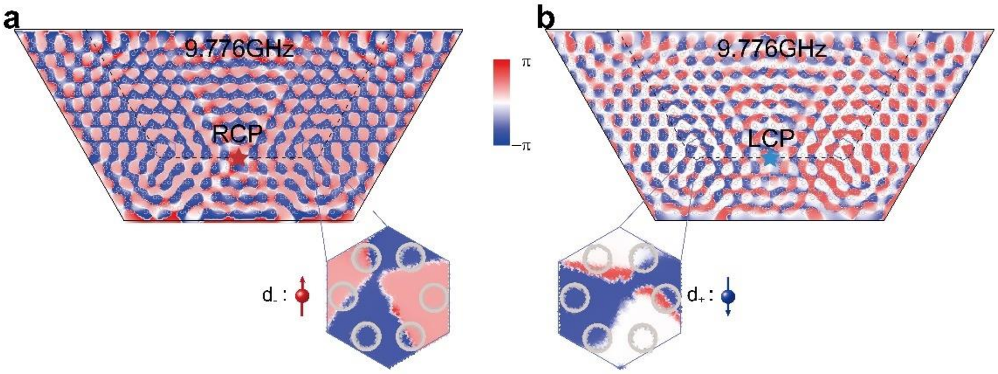

Supplementary Figure 12. | The eigenmode distribution of a finite-size tight-binding model. Bulk states, edge states, and corner states are represented by grey, yellow and blue dots respectively. There are two corner states which slightly split from the zero energy.

Supplementary Note 1. Band inversion induced by deforming the lattice.

Here we present the band structures for three different lattices: (1) the shrunken lattice (ordinary insulator, or OI). (2) the honeycomb lattice. (3) the expanded lattice (topological insulator, or TI) as shown in Supplementary Figure 1. These three band structures demonstrate a topological phase transition labeled by a band inversion process at the Brillouin zone center. For OI and TI lattices, the corresponding geometric parameters are the same as those in Fig. 2**a**. From Supplementary Figure 1, we find that the four-fold degenerate Dirac point is opened into two two-fold degenerate points consisting of two p and d states. Moreover, when we deforming the lattice from the shrunken lattice to the expanded lattice, p and d states are inversed as shown in Supplementary Figure 1, suggesting a topological phase transition.

Supplementary Note 2. Maximal Wyckoff positions and the primitive generator

The higher-order topology can be described by the fractional charges in $\mathrm{C_n}$ symmetric topological crystalline insulators (TCIs) [1]. We can determine the first-order and the second-order topological insulating phases by computing the position of the Wannier center in the unit cell for our photonic crystals. Owing to three mirror symmetries, the Wannier center is always located at the maximal Wyckoff positions as shown in Supplementary Figure 2**a**. There are three cases: (i) When the Wannier center is at the center of the unit cell, the photonic crystal is an atomic insulator which is always topologically trivial. (ii) When the Wannier center is at the center of the edge of the unit cell, the photonic crystal is a first-order topological insulator with topologically protected 1D edge states. (iii) When the Wannier center is at the corner of the unit cell, the photonic crystal is a second-order topological insulator with corner states. We can use a 2D tight-binding Hamiltonian to mimic our photonic crystals with $\mathrm{C_6}$ symmetry. This rotation symmetry puts non-trivial

constraints on the band structures. We can choose the eigenstates of the Hamiltonian at high symmetry points in the first Brillouin zone as common eigenstates of the six-fold rotation operator as following

$$\Pi_{\mathrm{p}} = e^{2\pi i(p-1)/6}, \text{ for } p = 1,2,3,4,5,6 \tag{1}$$

Next, we focus on corner physics. We know that the sum of two $\mathrm{C_n}$ symmetric TCIs is also a TCI with the rotation representation being the sum of two previous rotation representations. This kind of free Abelian additive structure of the TCI classification allows us to choose a set of primitive systems (hopping configurations of TCIs) to generate all TCIs up to a stable equivalence. We call these primitive systems primitive generators and for our photonic crystals, the corresponding primitive generators are $h_{3c}^{(6)}$ [1].

Supplementary Note 3. The $h_{4b}^{(6)}$ case of the C_6 symmetric lattice

Besides the $h_{3c}^{(6)}$ case considered above, there is another hopping configuration of C_6 symmetric lattice which is denoted as the $h_{4b}^{(6)}$ [1]. However, we do not consider it in this manuscript. The reason is explained as follows. For dielectric photonic crystals, except for the first band, the low-energy bands in the band structure matches well with the band structure of the tight-binding model (TBM) with nearest-neighbor coupling which has the same lattice structure. This is because, for the low-energy bands (the wavelength is in the same magnitude of the lattice constant), the couplings between the nearest neighbor lattice sites are induced by the coupling between the local Mie resonance modes of the dielectric rod where the nearest-neighbor coupling approximation is valid. As a consequence, the band structure of the photonic crystal in h_{3c} configuration matches well with the TBM. However, this is not the case for $h_{4b}^{(6)}$ lattice. As shown in Supplementary

Figure 3, although we arranged the lattice of dielectric rods in the form of $h_{4b}^{(6)}$ case, there is no full bandgap which is different from that of the TBM of $h_{4b}^{(6)}$ case. In $h_{4b}^{(6)}$ case of photonic crystals, the higher-order couplings such as the next-nearest-neighbor coupling have a significant influence on the band structure and cannot be ignored. Therefore, we cannot find the topological edge states as well as the corner states in the meta-structure as shown in Supplementary Figure3.

Supplementary Note 4. The ribbon structure

The ribbon structure used to calculate the projected band structure in Fig. 2**b** is shown in Supplementary Figure 4. We choose eight unit cells for each side of topologically distinct photonic crystals.

Supplementary Note 5. Eigenmodes in the hexagonal meta-structure

In the hexagonal meta-structure, due to the C_6 symmetry, there are six degenerate corner states in the bandgap between 1D edge states. Owning to the finite size effect, these corner states are linearly combined and form six near-degenerate states as shown in Supplementary Figure 5. From Fig. S5, we find that these six states are one monopole (denoted as s state), two degenerated dipoles (denoted as p state), two degenerated quadrupoles (denoted as d state) and one hexapole states (denoted as f state) respectively. We also measured these states by the near-field scanning method which matches the simulations well (see Supplementary Figure 5**b**).

As shown in Supplementary Figure 5 and Supplementary Figure 6, the field distribution far away from the source could be much higher than that from adjacent corners to the source. This can be explained as follows. In the hexagonal structure, boundaries between the HOTI and trivial insulator are closed. Therefore,

the electromagnetic wave is circling this closed-loop, forming whisper-gallery modes. Due to the precision of the fabrication in the experiment and meshing process in the finite-element calculation, there may be some nodes (waves at corners) that are brighter than other places that are irrelevant to the distance from the source.

Supplementary Note 6. Corner states in the tight-binding model

In our photonic crystals, dielectric rods are coupled by evanescent waves and thus photonic crystals share the same topological properties of the tight-binding model with only nearest-neighbor couplings [2]. The Hamiltonian is as follows

$$H = \begin{pmatrix} 0 & t_1 & 0 & t_2 e^{i\boldsymbol{k}(\boldsymbol{a_1}-\boldsymbol{a_2})} & 0 & t_1 \\ t_1 & 0 & t_1 & 0 & t_2 e^{-i\boldsymbol{k}\boldsymbol{a_2}} & 0 \\ 0 & t_1 & 0 & t_1 & 0 & t_2 e^{-i\boldsymbol{k}\boldsymbol{a_1}} \\ t_2 e^{i\boldsymbol{k}(\boldsymbol{a_2}-\boldsymbol{a_1})} & 0 & t_1 & 0 & t_1 & 0 \\ 0 & t_2 e^{i\boldsymbol{k}\boldsymbol{a_2}} & 0 & t_1 & 0 & t_1 \\ t_1 & 0 & t_2 e^{i\boldsymbol{k}\boldsymbol{a_1}} & 0 & t_1 & 0 \end{pmatrix} \tag{2}$$

Here t_1 and t_2 represent the intercell hopping and intracell hopping respectively. $\boldsymbol{a_1} = (a, 0)$ and $\boldsymbol{a_2} = (a/\boldsymbol{2}, \sqrt{\boldsymbol{3}}a/\boldsymbol{2})$ are two lattice vectors as shown in Supplementary Figure 7**a.** Here $\mathbf{k} = (k_x, k_y)$ represents the momentum. By diagonalizing this matrix, we can obtain the band structure.

If the intercell hopping is larger than the intracell hopping, the tight-binding model is a second-order topological insulator [2]. To mimic our implementation, we here consider a finite-size structure as shown in Supplementary Figure 7**b.** We solve the eigenmodes and find that there are zero-energy states in which the amplitude of eigenfunction is localized at the two corners as shown in Supplementary Figure 7**c.** The field distribution shares the same form as the corner states of our photonic crystals.

The eigenmode distribution of the finite-size tight-binding model in Supplementary Figure 7**b** is shown in Supplementary Figure 8. Due to the sublattice symmetry, the corner states are located at the zero-energy and are mixed with the edge states. A small energy shift of the corner states away from the zero-energy is induced by the finite-size effect. The field distributions of two corner states including the phases are presented in Supplementary Figure 9. We find that phase distributions of the corner states in the tight-binding model are similar to the corner states in our photonic crystals as shown in Fig. 3**c-d**.

Supplementary Note 7. Positions of the Measurement.

As shown in Supplementary Figure 10 and Fig. 3**a**, for the local field intensity of A/B is defined as the averaged value of experimental data of nine adjacent spatial points centered on the dielectric rods. For the other side of the corner, we measure in a mirror-symmetric way.

Supplementary Note 8. The measurement of the right corner spectrum

Besides the spectrum measured at the left corner, we also provide the spectrum measured at the right corner. As shown in Supplementary Figure 11, the spectrum measured at the right corner is almost the same as the one measured at the left corner. This is because the boundary is a closed loop in the hexagonal structure and the electromagnetic wave is circling inside this loop.

Supplementary Note 9. Phase distributions of all corner states

In this section, we provide the phase of all corner modes as shown in Supplementary Figure 12 from the numerical simulations. The phases distribution of corner states are constant (with a change of the sign) and have no vortex structure and singularity.

Supplementary References

[1] W.A. Benalcazar, T. Li and T.L. Hughes, Quantization of fractional corner charge in Cn-symmetric higher-order topological crystalline insulators. *Phys. Rev. B* **99**, 245151 (2019).

[2] Liu, F., Deng, H.Y. and Wakabayashi, K., Helical topological edge states in a quadrupole phase. *Phys. Rev. Lett.* **122**, 086804 (2019).
